# Drug-Associated Adverse Events and Their Relationship with Outcomes in Patients Receiving Treatment for Extensively Drug-Resistant Tuberculosis in South Africa

**DOI:** 10.1371/journal.pone.0063057

**Published:** 2013-05-07

**Authors:** Karen Shean, Elizabeth Streicher, Elize Pieterson, Greg Symons, Richard van Zyl Smit, Grant Theron, Rannakoe Lehloenya, Xavier Padanilam, Paul Wilcox, Tommie C. Victor, Paul van Helden, Martin Groubusch, Robin Warren, Motasim Badri, Keertan Dheda

**Affiliations:** 1 Lung Infection and Immunity Unit, Division of Pulmonology and UCT Lung Institute, Department of Medicine, University of Cape Town, Cape Town, South Africa; 2 Institute of Infectious Diseases and Molecular Medicine, University of Cape Town, Cape Town, South Africa; 3 Department of Infection, University College London Medical School, London, United Kingdom; 4 Sizwe Tropical Diseases Hospital, Sandringham, Johannesburg, South Africa; 5 National Health Laboratory Service and Division of Clinical Microbiology and Infectious Diseases, University of Witwatersrand, Johannesburg, South Africa; 6 Division of Infectious Diseases, Department of Medicine, Faculty of Health Sciences, University of the Witwatersrand, Johannesburg, South Africa; 7 DST/NRF Centre of Excellence for Biomedical TB Research/MRC Centre for Molecular and Cellular Biology, Stellenbosch University, Stellenbosch, South Africa; 8 Center for Tropical Medicine and Travel Medicine, Department of Infectious Diseases, Division of Internal Medicine, Amsterdam Medical Center, University of Amsterdam, Amsterdam, The Netherlands; 9 College of Medicine, King Saud Bin Abdulaziz University for Health Sciences, Riyadh, Saudi Arabia; Copenhagen University Hospital, Hvidovre, Denmark

## Abstract

**Background:**

Treatment-related outcomes in patients with extensively drug-resistant tuberculosis (XDR-TB) are poor. However, data about the type, frequency and severity of presumed drug-associated adverse events (AEs) and their association with treatment-related outcomes in patients with XDR-TB are scarce.

**Methods:**

Case records of 115 South-African XDR-TB patients were retrospectively reviewed by a trained researcher. AEs were estimated and graded according to severity [grade 0 = none; grade 1–2 = mild to moderate; and grade 3–5 = severe (drug stopped, life-threatening or death)].

**Findings:**

161 AEs were experienced by 67/115(58%) patients: 23/67(34%) required modification of treatment, the offending drug was discontinued in 19/67(28%), reactions were life-threatening in 2/67(3.0%), and 6/67(9.0%) died. ∼50% of the patients were still on treatment at the time of data capture. Sputum culture-conversion was less likely in those with severe (grade 3–5) vs. grade 0–2 AEs [2/27(7%) vs. 24/88(27%); p = 0.02]. The type, frequency and severity of AEs was similar in HIV-infected and uninfected patients. Capreomycin, which was empirically administered in most cases, was withdrawn in 14/104(14%) patients, implicated in (14/34) 41% of the total drug withdrawals, and was associated with all 6 deaths in the severe AE group (renal failure in five patients and hypokalemia in one patient).

**Conclusion:**

Drug-associated AEs occur commonly with XDR-TB treatment, are often severe, frequently interrupt therapy, and negatively impact on culture conversion outcomes. These preliminary data inform on the need for standardised strategies (including pre-treatment counselling, early detection, monitoring, and follow-up) and less toxic drugs to optimally manage patients with XDR-TB.

## Introduction

Over the last two decades the entity of multidrug resistant tuberculosis (MDR-TB i.e. resistance to at least isoniazid and rifampicin) has emerged. In 2008 there were approximately 440 000 cases of MDR-TB globally[1]. Between 5 to 10% of MDR-TB cases are thought to be due to extensively drug resistant tuberculosis (XDR-TB i.e. resistance to rifampicin, isoniazid, any fluoroquinolone and one of the 2^nd^ line injectable agents i.e. kanamycin, amikacin or capreomycin). MDR-TB and XDR-TB now threaten to destabilise TB control in several regions of the world including Africa, Eastern Europe, Russia, central Asia, India and China[2].

In high burden settings treatment outcomes of MDR-TB are disappointing with only ∼50% of patients successfully completing treatment[3]. Outcomes in XDR-TB are poorer. We and others have recently shown that, in contrast to low and intermediate burden settings[4], less that 20% of patients with XDR-TB culture-convert in high burden settings[5], [6]. Treatment options, because of the high grade of drug resistance are severely limited and the higher the total number of appropriate drugs used in a regimen the better the outcome[5]. Thus, treatment interruption due to any cause may potentially subvert successful outcome in patients with XDR-TB. Failure to identify and manage presumed drug-associated adverse events (AEs) may also have serious implications for patient perceptions about toxicity versus benefit, and thus may impact on compliance. Even in adherent inpatients, we and others have recently shown that AEs are common in patients with XDR-TB[5], [7].

However, data about the relationship between AEs and treatment-related outcomes in patients with drug-resistant TB are scarce. It is also unclear how the *M. tuberculosis* strain phenotype and host factors such as HIV co-infection impact on the frequency and severity of AEs, and associated clinical outcomes. Given that capreomycin modulates outcomes and is a vital backbone of most XDR-TB treatment regimens[6], the frequency of AEs to capreomycin and their temporal relationship to treatment initiation are of interest. Collectively, these data can inform on several aspects of management including the design and monitoring of treatment regimens for XDR-TB and formulating strategies to prevent treatment interruption, thus facilitating compliance and minimising treatment failure. To address these gaps in our knowledge and, in particular, to evaluate the association of AEs with outcomes we reviewed the case records of 115 patients treated for XDR-TB at three treatment centres in South Africa.

## Methods

### Study setting and participants

**Figure 1:**
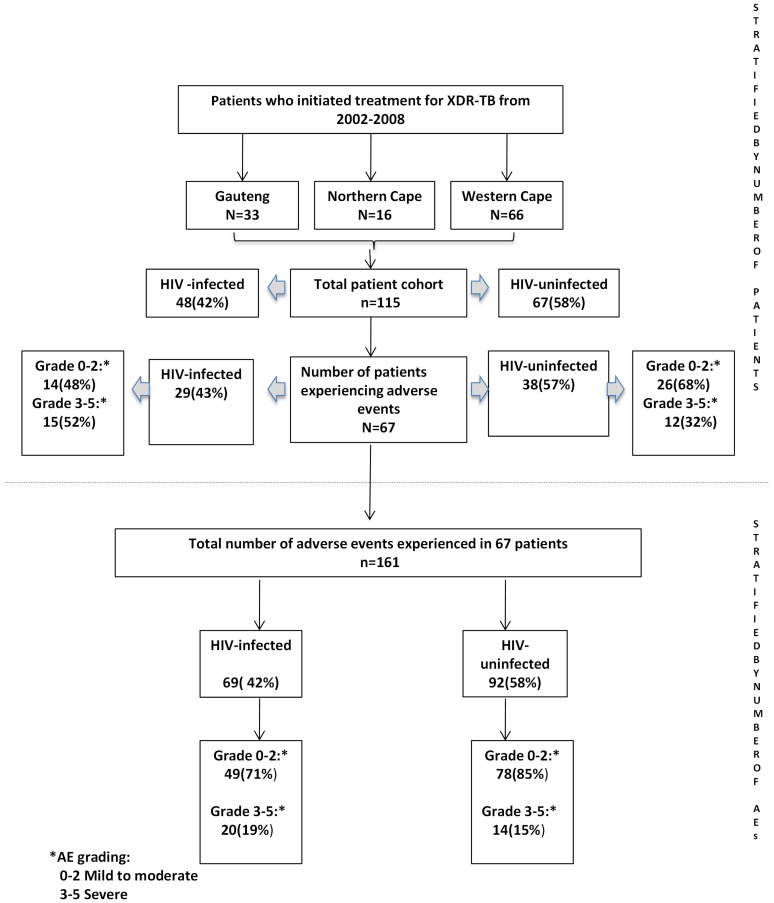
Study plan stratified according to treatment site, HIV status and severity of adverse drug reactions.

We retrospectively reviewed the case records of 115 consecutive laboratory-confirmed XDR-TB patients diagnosed between August 2002 and February 2008 at three designated XDR-TB treatment centres in South Africa (see Figure 1 for the study outline). Patients were admitted to the facilities for the duration of their treatment and thus adherence was assumed to be excellent unless the patients self discharged (designated as default from treatment). Case records were comprehensively reviewed by a trained researcher for AEs listed in Table 1 (including duration, type and severity), drug regimen used (dose, indication, route of administration), culture conversion and mortality outcomes, and HIV status. Associated demographic and clinical information were also transcribed into a case record form, and the information captured by double data entry.

**Table 1:** Definitions used to grade, identify and classify adverse events.

**A. Grading of adverse events** 1	
grade 0	no AE
grade 1	mild AE i.e. described in the patient's management records but no action was taken
grade 2	moderate AE resulting in either changing the dose or frequency of the offending drug or another drug(s) was added to manage the AE
grade 3	the side effect was severe enough for the offending drug to be stopped
grade 4	the AE was life threatening or disabling
grade 5	the AE caused the death of the patient
**B. Definitions used to identify and classify adverse events.**	
Nausea, vomiting, diarrhoea. Other GI symptoms: abdominal pain, dyspepsia, and epigastric discomfort	As documented by the physician or nursing staff
Dizziness/disorientation/confusion	
Body aches/pains/cramps	
Headache	
Sore tongue/throat	
Generalised itchiness	
Fatigue	
Numbness of extremities	Symptoms and findings consistent with neuropathy, e.g. pain or numbness of the distal extremities diagnosed by a physician.
Skin reaction	A dermatological reaction felt to be related to anti-tuberculosis medications as documented by the physician or dermatologist
Hypokalaemia	<3.5 meq/L (normal range: 3.5–5.5 meq/L)
Hypothyroidism	At least one thyroid stimulating hormone (TSH) result >4.94 IU/ml (normal 0.35–4.94) that was thought to be unrelated to the sick euthyroid syndrome
Depression/psychosis	As diagnosed by the TB physician and/or psychiatrist, based on international classification of diseases (ICD)-10 criteria
Visual disturbance	Diagnosed by the physician/eye specialist as being related to the TB drugs
Arthralgia	Painful joints as reported by patient and documented by physician or nurse
Ototoxicity	Hearing loss confirmed by audiometry and/or physical examination
Renal impairment/renal failure	Creatinine >100 µmol/L
Hepatotoxicity	Raised bilirubin or elevated transaminases >3 times the upper limit of normal, and ascribable to a specific drug

1 These were graded according to the modified American National Institute of Health common terminology criteria for adverse events [CTCAE].

### Ethics

Ethical approval was provided by the UCT Research Ethics Committee. As per the regulations at UCT patient-provided written informed consent was not required as retrospective annonymised data was used in this study.

### Diagnosis and treatment regimens

The standard definition of XDR-TB was used to define patient inclusion[8]. Drug-susceptibility testing to capreomycin, terizidone/cycloserine and fluoroquinolones other than ofloxacin was unavailable as these tests are not undertaken by the National Treatment Program (NTP). The drugs used in the treatment regimens are shown in Table 2. XDR-TB treatment was only initiated and administered in hospital with the use of capreomycin and para-aminosalycilic acid (PAS) as the anchor drugs since late 2006/early 2007. Treatment with capreomycin was empiric and in almost all cases was given in the absence of prior susceptibility testing. Third-line drugs (clarithromycin, dapsone, amoxicillin/clavulanate and azithromycin) were used at the discretion of the attending clinician. High-dose INH was administered at a dose of 10 mg/kg. Clofazimine and moxifloxacin was used in selected centres on a limited basis. ART was offered to all HIV co-infected patients irrespective of the patients' CD4 count.

**Table 2:** Specific drugs, the dosages used in XDR-TB treatment regimens, and the frequency of drug withdrawal due to adverse events relative to the number of patients prescribed the drug.

	Drug dosages used	No. of patients who received a drug as part of the XDR-TB regimen n = 115(%)	Number of patients in whom the drug was withdrawn relative to the total number receiving the drug (%)	Proportion of severe AE [total = 34] due to a specific drug (%)
Isoniazid	4–6 mg/kg/daily	39/115(34)		-
Ethambutol	25 mg/kg/daily	46/115 (40)	1/46(2.2)	1/34(2.9)
Pyrazinamide	30–40 mg/kg/daily	80/115(69.6)	-	-
Amikacin	15–20 mg/kg/daily*	3/115(2.6)	1/3 (33.3)	1/34(2.9)
Kanamycin	15–20 mg/kg/daily*	4/115(3.5)	-	-
Ofloxacin	600–800 mg daily	29/115(25.2)	-	-
Moxifloxacin	400 mg daily	2/115(1.7)	-	-
Ethionamide	15–20 mg/kg/daily	66/115(57.3)	7/66(10.6)	7/34(20.6)
Capreomycin	15–20 mg/kg/daily*	104/115(90.4)	14/104 (13.5)	14/34(41.2)
Para-aminosalicylic acid	8 g (400 mg BD)	101/115(87.8)	7/101(6.9)	7/34(20.5)
Terizidone/Cycloserine	500–750 mg daily	104/115(90.4)	2/104(1.9)	2/34(5.9)
Clarithromycin	1 g (500 mg BD)	77/115(66.9)	-	-
Amoxicillin-clavulanate	375 mg	65/115(56.5)	2/65(3.1)	2/34(5.9)
Clofazimine	200 mg (100 mg BD)	28/115(24.3)	-	-
Dapsone	100–200 mg daily	36/115(31.3)	-	-
Azithromycin	500 mg 3xweekly	11/115(9.6)	-	-
INAT (INH+thiacetazone)	3 tabs daily	2/115(1.7)	-	-
Rifabutin	300 mg daily	1/115(0.87)	-	-
**Type of ART**	**Dosage used**	**Number of XDR-TB patients receiving drug**	**Number of HIV-infected persons receiving ART**	
		**n = 115 (%)**	**n = 34 (%)**	
3TC (Lamivudine)	80 mg (40 mg BD)	29/115 (25)	29/34 (85.3)	-
D4T (Stavudine)	300 mg (150 mg BD)	25/115 (22)	25/34 (73.5)	-
EFV (Efavirenz)	600 mg nocte	25/115 (22)	25/34 (73.5)	-
NVP (Nevirapine)	200 mg BD	4/115 (3)	4/34 (11.8)	-
AZT (Zidovudine)	600 mg (300 mg BD)	5/115 (4)	5/34 (14.7)	-
Lopinavir/Ritonavir	800 mg (400 mg BD)	1/115 (1)	1/34 (2.9)	-

* (Maximum dose, 1 g) 5 days/week.

### Definition of adverse drug reactions

For the purposes of analysis grades 1 and 2 AEs were considered mild to moderate, and grade 3–5 severe (see definitions in Table 1). Events where the drug was discontinued was designated grade 3. Multiple events of the same AE were counted separately.

### Outcomes

All cause mortality and culture conversion were the primary outcomes of interest. Conversion was judged to have occurred when two consecutively negative cultures were obtained, 1 month apart, and providing that a culture taken at initiation of XDR-TB treatment was positive. Time to conversion was measured in days from treatment start date to the take date of the first of the two negative cultures.

### Mycobacterium tuberculosis strain typing

A subset of 53 XDR-TB isolates from patients from the Western Cape were genotyped by IS*6110* DNA fingerprinting [9] and spoligotyping [10]. Strains were categorised as Beijing or non-Beijing according the their spoligotype signature [11].

### Data handling and statistical analysis

A data risk management tool, including double data entry, was used to ensure data integrity. Categorical data were compared using χ^2^ test and continuous data were compared using Mann-Whitney test or Kruskal-Wallis test (SPSS, Version 17). Cox proportional hazards regression models were fitted to determine risk factors associated with outcomes in a time-to-event (conversion and mortality) based analysis. These factors included AEs, previous MDR-TB, 6 month culture conversion (when assessing risk factors for death), HIV status, usage of ART, weight, age, sex, ethnicity, number of previous TB episodes, number of drugs used in a regimen. Factors found to be significant in univariate were included in the final multivariate analysis. Kaplan-Meier's method was used to calculate probabilities of events, and the Log-rank test was used to compare these probabilities by group. All tests were two-sided, and a p-value <0.05 was considered significant. The proportionality assumption of the Cox models was tested using –ln[–ln (survival)] curves and regression of scaled Schoenfeld residuals on functions of time.

## Results

### Demographic and clinical characteristics

AEs were reported in 58.3% (67/115) of patients. The breakdown by severity of AE and HIV status, stratified by number of patients and total number of AEs, is shown in Figure 1. The median CD4 count in HIV-infected persons was 204 (range 13–893) cells/mm^3^. We could not identify any demographic and clinical variables that were specifically associated with the development of AEs (grade 1 to 5) compared to those who did not develop an AE (grade 0). However, patients with severe AEs (grade 3 to 5), when compared to those with mild, moderate or no AEs (grade 0, 1 and 2), were more likely to be female, have had previous MDR-TB or drug sensitive TB, and had fewer drugs in their treatment regimens (Table 3). Furthermore, in the multivariate analysis only a history of previous MDR-TB was independently associated with the likelihood of developing severe AEs (grade 3 to 5); p = 0.009. The overall median (IQR) duration of follow-up (months) within the cohort was 7.3 (3.1–12.6). As at the study censure date 21% of patients had died, 22% had defaulted treatment, 7% were cured or had completed treatment, and the remaining of 50% were on on-going treatment.

**Table 3:** Socio-demographic and treatment related clinical characteristics of 115 patients who initiated treatment for extensively drug-resistant tuberculosis (XDR-TB).

	Grade: 0–2 (none/mild/moderate) adverse events	Grade 3–5 (severe/life threatening/death) adverse events	P value
	n = 88 (% unless otherwise stated)	n = 27 (% unless otherwise stated)	
**Sex**			
male	53(60.2)	9(33.3)	0.014
**Ethnicity**			
mixed origin	46(52.3)	14(51.9)	0.969
**HIV status**			
infected	33(37.5)	15(55.6)	0.096
**ART**			
yes	23(71.8)	11(73.3)	0.917
**Number of previous sensitive TB episodes (IQR)**	1(1–2)	1(1–2)	0.033
**Previous MDR-TB episodes**			
yes	47(53.4)	22(81.5)	0.009
**Previous MDR-TB episodes (IQR)**	1(1–1)	1(1–2)	0.467
**Number of drugs in the treatment regimens (IQR)**	6(5–7)	5(4–6)	0.001
**Smoking**			
current	37(42)	12(44.4)	0.454
non	36(40.9)	13(48.1)	
previously	15(17)	2(7.4)	
**Weight at diagnosis** of XDR-TB (IQR)	48(44–59)	48(36–59)	0.626
**Age at diagnosis** of XDR-TB (range in years)	31.0(26.4–42.0)	36.8(25.0–46.2)	0.892
**Outcome-related variables**			
**Died**			
yes	17(19.3%)	13(48.1%)	0.003
**Conversion**			
yes	24(27.3%)	2(7.4%)	0.035

* The only other ethnic group in the cohort was Black. Grade 0–2 AE 42(47.7%). Grade 3–5 13(48.1%).

### Frequency and severity of AEs

161 AEs were experienced by 67/115 (58%) patients (Figure 1; upper panel). When the results were stratified by the number of patients 17/67 (25.4%) patients required no intervention (grade 1); 23/67 (34.3%) required modification of treatment (grade 2), the offending drug was discontinued in 19/67 (28.3%) of patients (grade 3); reactions were life-threatening in 2/67(3.0%; grade 4), and 6/67(9.0%) died (grade 5). When the results were analysed by the number of AEs (Figure 1; lower panel): in 58/161 (36%) instances an AE was described but there was no intervention; 69/161 (43%) required modification of treatment in either the dose or frequency of the drug being taken, or, the prescription of an additional drug to treat the AE; the offending drug was withdrawn in 34/161 (21%); the AE was life-threatening in 2/161 (1.2%) instances (both AEs were due to renal failure), and death was associated with 6/161 (4%) of AEs. All 6 deaths were associated with capreomycin (hypokalaemia in 1 patient and renal failure in 5 others), and these patients died at a median of 14 days (range of 9–73 days) after starting therapy including Capreomycin. The severity of AEs was not associated with the frequency and duration with which the drug was used, or the resistance pattern of the drug.

### Culture conversion and mortality outcomes stratified by HIV status

**Figure 2:**
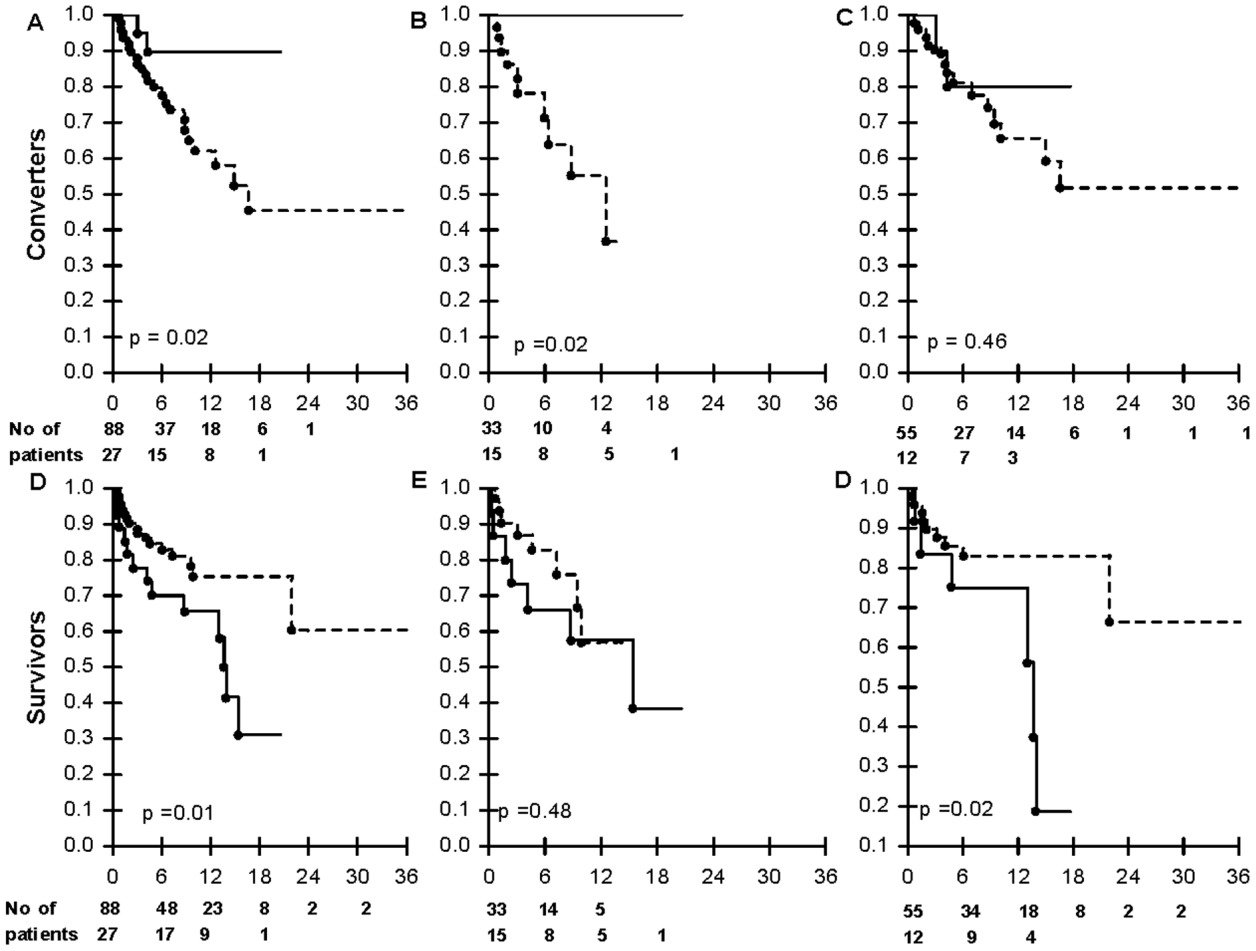
Kaplan-Meier probabilities of XDR-TB culture-conversion in: (A) The whole cohort of patients who experienced AEs stratified by severity score i.e. none or mild to moderate (grade 0, 1 and 2; dashed line) versus severe (grade 3 to 5; solid line); (B) HIV-infected patients whom experienced AEs stratified by stratified by severity score; (C) HIV-uninfected patients who experienced AEs stratified by stratified by severity score, and Kaplan-Meier probabilities of death: (D) The whole cohort of patients from the date of treatment-initiation, (E) HIV-infected patients who experienced AEs stratified by severity score, and (F) HIV-uninfected patients who experienced AEs stratified by severity categories.

Culture conversion occurred in 26/115 (22.6%) of patients. Patients with grade 3–5 AEs had a lower sputum culture conversion rate compared with those with grade 0–2 AEs [2/27 (7.4%) vs. 24/88 (27.3%); p = 0.02; Figure 2A]. In a Cox regression of the whole cohort the hazard ratio for AE (grade 3–5 AEs compared with grade 0–2 AEs) as a risk factor for culture conversion was 0.22 (0.05–0.95); p = 0.04. There were no other significant variables associated with culture conversion.

In contrast to HIV-uninfected patients (Figure 2C), HIV-infected patients (Figure 2B) with severe AEs (grade 3–5) had a significantly lower sputum culture conversion rate than those with grade 0–2 AEs [0/15 (0%) vs. 10/33 (30.3%), p = 0.02].

Of the 115 patients in the cohort, 30 (26.1%) died. Patients with grade 3–5 AEs had a higher death rate compared with those with grade 0–2 AEs [13/27 (48.1%) vs. 17/88 (19.3%); p = 0.003; Figure 2D]. However, in a multivariate Cox regression model for risk factors for death in the whole cohort, only culture non-conversion and previous MDR-TB, but not adverse events, were independently associated with death (Table 4).

**Table 4:** Univariate and multivariate analysis of factors associated with mortality in 115 patients with XDR-TB.

Factor	Univariate analysis		Multivariate analysis	
	Hazard Ratio (95%CI)	P-value	Hazard Ratio (95%CI)	P-value
Adverse event				
Grade 3–5	2.39(1.14–4.97)	0.02	1.43(0.67–3.05)	0.35
Grade 0–2	1		1	
Previous MDR TB				
Yes	3.27(1.32–8.03)	0.01	2.91(1.16–7.35)	0.02
No	1		1	
6 month Culture conversion				
Yes	0.09(0.01–0.63)	0.02	0.10(0.01–0.747)	0.03
No	1		1	

In HIV-infected patients mortality rates were higher in those with grade 3–5 (severe) AEs compared to those with grade 0–2 AEs [7/15 (46.7%) vs. 8/33 (24.2%); p = 0.12; Figure 2D, 2E, 2F]. Similarly, in the HIV-uninfected patients, those with severe AEs had a higher death rate compared to those without severe AEs [6/12 (50.0%) vs. 9/55 (16.4%); p = 0.02; Figure 2F]. Of the 13 all-cause deaths occurring in the severe AE group, 6 were due to an AE itself (5 due to renal failure and 1 due to hypokalaemia-all likely ascribable to capreomycin). These patients were not terminally or critically ill and there was a clear temporal relationship between the initiation of the drug and the patient's death. Five out of the 6 patients who died from AEs were HIV-infected.

25/115 (21.7%) of patients defaulted (self discharged) from the inpatient facilities. There was no difference between the proportion of patients with severe AEs among defaulters and the proportion with severe AEs among non-defaulters [7/25 (28%) vs. 27/90 (30%); p = 0.96].

### AEs by HIV status

In HIV-infected versus uninfected persons there was no significant difference between the proportion of persons with AEs [29/48 (60.4%) vs. 38/67 (56.7%); p = 0.26)], the number of total AEs per person [2.37 vs. 2.42 AEs per person; p = 0.15], and the number of severe AEs [20/69(29.0%) vs. 14/92(15.2%); p = 0.31]. Thus, the type, frequency and severity of the number of AEs was similar in HIV-infected and uninfected patients. However, those who died of an AE were more likely to be HIV-infected than HIV un-infected [5/6 (83.3%) vs. 1/6 (16.7%), p = 0.01)].

34/48 (71%) HIV-infected patients were on ART (active anti-retroviral therapy). 23/34 (68%) of patients were on a combination of lamivudine (3TC), stavudine (D4T) and efavirenz (EFV). In HIV-infected patients the number of patients experiencing an AE was not significantly different in those taking ART vs. those not taking ART [29/34 (85.3%) vs. 8/14 (57.1%); p = 0.71]. Similarly, the frequency of severe AEs was not significantly different in the same groups [11/34 (32.3%) vs.6/14 (43.0%)]. Thus, ART did not impact on the frequency of AEs and was generally well tolerated. The role of overlapping toxicities between ART and anti-TB drugs could not specifically be evaluated but the number of patients experiencing an AE was significantly higher in those taking ART compared to HIV un-infected patients [29/34 (85.3%) vs. 38/67 (56.7%); p = 0.008]. Nevertheless, the proportion of patients experiencing a severe AE was not significantly different in those taking ART compared to HIV un-infected patients [11/34 (32.3%) vs. 12/67 (17.9%); p = 0.17].

### Type of adverse events and drug withdrawal

Of all drug discontinuations, (n = 34), capreomycin (Capstat; Pharmacare Johannesburg) was the drug withdrawn most often in 14/34 (44.1%) of cases, followed by PAS in 7/34 (20.5%), and ethionamide in 7/34 (20.6%) (Table 2). The withdrawal of capreomycin due to an AE occurred at a median of 73 days (range 9–485) days after initiation of therapy. Persons who took capreomycin and had AEs, compared to those that took capreomycin but had no AEs, were more likely to be taking concurrent ethambutol, augmentin, ethionamide and PZA (p<0.05).

The breakdown of AEs by cause is shown in Table 5. Overall, nausea and vomiting (22%), diarrhoea (14%), and other GI symptoms (14%) were the commonest causes of AEs and their frequency did not differ significantly by HIV status. The most common cause of grade 0–2 AEs (79% of the total number of AEs) were GI symptoms overall (nausea, vomiting, diarrhoea and others), which caused ∼50% of AEs in this severity category. The most common causes of grade 3–5 AEs (21% of the total number of AEs) were specifically vomiting (29% of severe AEs) and renal failure (21%).

**Table 5:** Type of adverse event that occurred (n = 161) and total number of patients experiencing these adverse events (n = 115) in patients from the Western Cape, Northern Cape and Gauteng provinces who initiated treatment for XDR-TB.

Presumed drug-associated adverse event	Number of AEs stratified by HIV status (n = 161)			Number of patients with AEs stratified by HIV status (n = 115)		
	n (%)			n (%)		
	HIV+(69)	HIV- (92)	Total AE (161)	HIV+(48)	HIV- (67)	Total patients (115)
Nausea and/or vomiting	15(9)	20(12)	35 (22)	15(31)	20(30)	35(30)
Diarrhoea	8(5)	14(9)	22 (14)	8(17)	14(21)	22(19)
Other GI symptoms: abdominal pain, dyspepsia, epigastric discomfort, cramps	6 (4)	16(10)	22 (14)	6(13)	16(24)	22(19)
Dizziness/disorientation	4(2)	9(6)	13 (8)	4(8)	9(13)	13(11)
Hearing loss	2(1)	8(5)	10 (6)	2(4)	8(12)	10(9)
Renal failure	4(2)	3(2)	7 (4)	4(8)	3(4)	7(6)
Body aches/cramps	5(3)	5(3)	10 (6)	5(10)	5(7)	10(9)
Headache	6(4)	2(1)	8 (5)	6(13)	2(3)	8(7)
Skin reaction	3(2)	4(2)	7 (4)	3(6)	4(6)	7(6)
Hypokalaemia	5(3)	2(1)	7 (4)	5(10)	2(3)	7(6)
Hypothyroidism	3(2)	3(2)	6 (4)	3(6)	3(4)	6(5)
Depression	1(1)	1(1)	2 (1)	1(2)	1(1)	2(2)
Sore tongue/throat	1(1)	1(1)	2 (1)	1(2)	1(1)	2(2)
Numbness of extremities	2(1)	0	2 (1)	2(4)	0	2(2)
Generalised itchiness	1(1)	1(1)	2 (1)	1(2)	1(1)	2(2)
Psychosis	0	1(1)	1(1)	0	1(1)	1(1)
Renal impairment	1(1)	0	1(1)	1(2)	0	1(1)
Fatigue	0	1(1)	1(1)	0	1(1)	1(1)
Visual disturbance	1(1)	0	1(1)	1(2)	0	1(1)
Thrombophlebitis	0	1 (1)	1(1)	0	1(1)	1(1)
Arthralgia	1(1)	0	1(1)	1(2)	0	1(1)
Total # AE stratified by HIV Status	69(43	92(57)	161(100)			

Some patients experienced multiple AEs. These were frequently clustered in the gastro-intestinal subgroup. Thus, of those who had diarrhoea 15/22 (68.2%) also experienced nausea and vomiting, and 10/22 (45%) nausea and vomiting together with abdominal pain and dyspepsia. AE clustering was also evident in the neurological category (overlapping symptoms of headaches, dizziness, generalised aches and pains etc.).

For the 18 drugs used in the XDR-TB treatment regimens, the severity of AEs was not related to the number of patients who received each drug, total duration of treatment (months), or the proportion of resistant isolates.

### AEs stratified by *Mycobacterium tuberculosis* strain type

Of the 115 patients with XDR-TB, isolates were available for genotyping in 53 of the patients from the Western Cape. Significantly more patients had a Beijing compared to a non-Beijing strain [43(81%) vs. 10 (1%); p = 0.0001]. The severity of AEs was not significantly different in the Beijing and non-Beijing families (Table 6).

**Table 6:** Effect of TB strain type on AEs stratified by Beijing and non-Beijing strain type.

Strain type	Beijing		Non-Beijing	
	n(%)		n (%)	
**Severity of AE**	AEs 0–2	AEs 3–5	AEs 0–2	AEs 3–5
**HIV-infected**	9/14	5/14	1/3	2/3
	(64.3)	(35.7)	(33.3)	(66.7%)
**HIV-uninfected**	20/29	9/29	5/7	2/7
	(69.0)	(31.0)	(71.4)	(28.6)
**Sub-totals**	**29/43**	**14/43**	**6/10**	**4/10**
	**(67.4)** *	**(32.6)** *	**(60)**	**(40)**
**Total**	**43/53(81)** **		**10/53(19)** **	

* p = 0.03 (severe vs. mild to moderate AEs).

** p = 0.0001 (total Beijing versus non-Beijing).

## Discussion

This is the first comprehensive report of AEs and their association with outcomes in a large cohort of patients with XDR-TB. Our key findings were that: (i) the frequency of AEs with XDR-TB treatment regimens is high (∼60%), and in ∼40% of patients the AE was associated with interruption of therapy, life-threatening reactions, or fatal consequences; (ii) those who died of an AE were more likely to be HIV-infected and thus greater vigilance is required in this group; (iii) those with severe AEs have poorer culture conversion but not higher mortality underscoring the need for careful treatment monitoring for early detection of AEs, and (iv) capreomycin was likely the most common cause of drug withdrawal (44% of all withdrawals), was likely responsible for over 40% of severe AEs and all AE-related deaths, and thus careful monitoring of this drug is mandatory.

A fundamental finding of this study is that XDR-TB patients with severe AEs had poorer culture-conversion outcomes. By contrast, in patients with MDR-TB from Turkey[12] and Russia where AEs were common (∼70% of patients) AEs were not associated with unfavourable outcomes[13]. Thus, in contrast to MDR-TB, in XDR-TB patients the consequences of AE-associated interruption of individual drugs impacts on culture-conversion outcomes. This most likely reflects discontinuation of crucial drugs like capreomycin. Thus, interruption of drug therapy has deleterious consequences. In keeping with the findings of O'Donnell *et al* [7] we found no association between AEs and mortality in the multivariate analysis.

In our study persons who took capreomycin and had AEs, compared to those that took capreomycin but had no AEs, were more likely to be taking concurrent ethambutol, augmentin, ethionamide and PZA, raising the possibility that capreomycin withdrawal in some cases may have been unwarranted. However, these confounding drugs are rarely a cause of renal failure and are not associated with hypokalemia. The high capreomycin toxicity seen in our study (almost half of all drug withdrawals due to an AE) is in keeping with the findings of a Peruvian study where 31% of 115 MDR-TB patients had hypokalaemia, which was independently associated with the administration of capreomycin[14]. Based on our findings we suggest weekly checks of renal function and electrolytes in the 1^st^ 4 weeks of therapy, and then every 2 weeks for the next two months, and monthly thereafter. Our data also raises the question of routine supplementation of electrolytes in patients on capreomycin treatment, and capreomycin drug susceptibility testing in all patients with suspected or proven XDR-TB. We suggest active monitoring for AEs, correct dosing by body weight, correction of dehydration, and regular monitoring of renal function and electrolytes, particularly in those with risk factors (hypertension, diabetes, HIV-associated nephropathy, vomiting and diarrhoea, dehydration, electrolyte abnormalities, diuretic usage, alcohol abuse, and use of potentially nephrotoxic drugs such as tenofovir, cotrimoxazole). This has implications for the out-patient management of XDR-TB, which is currently being rolled out in high burden settings due to the sheer burden of cases that have overwhelmed designated facilities[4]. Our data inform on resource allocation by national TB programmes in high burden settings that will need to take into account provision of monitoring and laboratory infrastructure when planning decentralised and nurse-led services for drug-resistant TB. Given the associated poorer outcomes in XDR-TB patients with AEs, health care workers should be educated about the recognition, management, as well as appropriate referral pathways of those experiencing AEs, and patients should be followed up more closely and offered appropriate counselling to ensure drug adherence. Our recommendations are easily implementable and do not detract from providing decentralised MDR treatment services in resource-poor settings.

Nausea and vomiting, in keeping with the findings of Shin et al.[13], was the most common reason for discontinuation of drug therapy (in any severity category) and needs to be managed with patient counselling, anti-emetics, and/or splitting of the dose to improve tolerability. AEs although frequent were not more common in HIV-infected patients unlike observations that we[15] and others[16] have documented in patients with drug-sensitive TB. The reasons for this are unclear but could reflect poorer absorption of second line drugs and hence lower serum levels, or, be an ascertainment bias as HIV-infected patients may have died prior to diagnosis. Nevertheless, HIV-infected patients were more likely to die from severe AEs and increased vigilance and correct dosing by body weight is required in this group.

The frequency of AEs in this study (∼60%) are similar to those that evaluated AEs to second line drugs in the context of XDR-TB (58%)[7] and MDR-TB (73.3% in Tomsk, Russia[13] and 69,2% in Istanbul Turkey[12]) but twice that of AEs to first line drugs in those with drug-sensitive TB[17]. Suspension of any agent (28% in our study) occurred at a similar frequently compared to a large multi-centric study in patients with MDR-TB (30%)[18] and in patients with XDR-TB[7], more frequently in a Peruvian study in patients with MDR-TB (14%) [19], but less frequently than in Turkish patients with MDR-TB (55%)[12]. This may reflect the heterogeneity of several factors including HIV rates, previous history of TB of any type, resistance profiles, drug regimens, physician management and ascertainment bias.

We found no significant association between strain genotype and the frequency or severity of AEs. This may reflect a true lack of association or type 2 error given the small numbers of isolates that were accessible for genotyping. Association with strain type is of interest because Beijing strains are thought to be more virulent (more cavitation and greater disease extent) and such patients may require a prolonged injectable phase and an increased number of drugs in a regimen. Moreover, recent data suggest that DR-TB strains have, in addition to resistance conferring mutations, hundreds of compensatory mutations that may alter the structure and hence antigenic properties of the organism[20]. This may impact host immune profiles and hence interaction with drug compounds. Further and larger studies are required to clarify this issue.

Similar to findings in earlier studies in drug-sensitive TB[16], [17], [21], a higher number of women experienced AEs. The reason for this remains unclear. Similar to the findings in the context of drug-sensitive TB[21], the higher rate of AEs in those with prior MDR-TB may reflect prior sensitisation, higher drug levels in patients with a lower body weight, and the generally poorer health status in keeping with chronic disease.

There are several limitations of our study. These include the retrospective study design, lack of complete adherence data, ascertainment bias due to retrospective data capture from medical notes, physician bias, use of a single researcher to capture data on a standardised template, inability to calculate drug-specific AE rates and AE rates per person months of exposure, or to definitively delineate AEs from disease-related morbidity in HIV-infected patients. However, this is difficult to calculate even in prospective studies because of the inability to ascribe a particular AE to a specific drug in a multidrug regimen. Thus, we chose the term adverse event (rather than adverse drug reaction) as in some cases it was impossible to ascribe the event to a drug rather than HIV, and in other instances it was impossible to determine whether it was TB drug or ARV-related, and in each of these cases which specific drug was implicated. Nevertheless, the patients were consistently seen by a small group of experienced clinicians who based assessments on their clinical judgement and temporal relationship to symptoms, signs, and laboratory data, and we only extracted variables that could be confidently ascertained. We were also reliant on the judgement and investigative evaluation of clinicians who ascribed renal failure to capreomycin rather than dehydration, vomiting and diarrhoea etc., and we could only captured events ascribed by a clinician to be significant. Thus, our analysis may reflect this clinical bias. Given that DST for capreomycin was unavailable, we may have in many, or possibly the majority of cases, inappropriately treated with capreomycin and hence over-estimated the magnitude of AE. However, DST for capreomycin is unreliable and clinical benefit may still occur, even in the face *in-vitro* resistance, and cross-resistance between capreomycin and amikacin is greater than for kanamycin, which is used in our treatment programme. A further limitation is that although all the patients were hospitalised and drugs administered strictly on a DOT basis, it remains unclear to what extent patients may have circumvented this process, and this could have confounded our findings. Furthermore, we did not capture the pill burden or its relationship to the frequency and severity of adverse events. Survivor selection bias may have led to an underestimate of the true mortality in the HIV-infected sub-group whilst late detection and delayed management of AEs could have contributed to mortality given that severe AEs occurred on average about 2 months after mild to moderate AEs. Only a prospective study will be able to address this hypothesis. There were few events in the group with severe AEs and thus CIs were wide, and larger studies are needed to confirm our findings. Finally, our findings are only generalisable to a resource-poor high HIV prevalence setting like South Africa where there is a high rate of prior MDR-TB.

In conclusion, the frequency of AEs with XDR-TB treatment regimens is high and often severe. Those with severe AEs have poorer treatment-related outcomes. Early detection and monitoring of AEs is thus crucial, and XDR-TB patients with AEs should be closely monitored for the remainder of their therapy. Assays to monitor serum levels of second line drugs and less toxic drugs are urgently needed. These data inform on the management and monitoring of patients being treated for XDR-TB, factors that impact on patient compliance, and the provision of resources within national TB programmes that seek to offer decentralised and nurse-led care for patients with XDR-TB.
