# Correction: Drug-Associated Adverse Events and Their Relationship with Outcomes in Patients Receiving Treatment for Extensively Drug-Resistant Tuberculosis in South Africa

**DOI:** 10.1371/annotation/644591a8-8ae6-450e-974e-1cd1f08f52c7

**Published:** 2013-05-31

**Authors:** Karen Shean, Elizabeth Streicher, Elize Pieterson, Greg Symons, Richard van Zyl Smit, Grant Theron, Rannakoe Lehloenya, Xavier Padanilam, Paul Wilcox, Tommie C. Victor, Paul van Helden, Martin P. Grobusch, Robin Warren, Motasim Badri, Keertan Dheda

The name of the twelfth author should be: Martin P. Grobusch 

